# Modulation of neural complexity and consciousness in temporal lobe seizures: Effects of high‐frequency pulvinar stimulation

**DOI:** 10.1002/epd2.70056

**Published:** 2025-06-21

**Authors:** Ionuț‐Flavius Bratu, Nada El Youssef, Stanislas Lagarde, Fabrice Bartolomei

**Affiliations:** ^1^ Department of Epileptology and Cerebral Rhythmology Timone Hospital Marseille France; ^2^ Aix‐Marseille University, French National Institute of Health and Medical Research (UMR 1106), Systems Neuroscience Institute Marseille France

**Keywords:** consciousness, permutation entropy, pulvinar, SEEG, stimulation

## Abstract

**Background:**

Loss of consciousness/awareness during temporal lobe seizures significantly affects quality of life and is closely linked to pathological thalamocortical synchronization and loss of cortical signal complexity. The medial pulvinar nucleus (PUM) contributes to seizure propagation and awareness impairment, making it a potential target for neuromodulation. Acute high‐frequency PUM stimulation has previously been shown to reduce seizure severity and improve awareness, potentially by disrupting excessive synchrony.

**Aims:**

In this study, we investigated the effects of PUM stimulation on signal complexity and its relationship to ictal awareness using permutation entropy (PE) in SEEG recordings.

**Materials and methods:**

Eight patients with focal drug‐resistant temporal epilepsy underwent hippocampus‐induced seizures with and without additional high‐frequency PUM stimulation. SEEG complexity changes were quantified using PE and ictal awareness was assessed using the Consciousness Seizure Scale (CSS).

**Results:**

Our results showed that PUM stimulation attenuated entropy reductions during seizures, suggesting a preservation of neural complexity. Moreover, reduced entropy alterations correlated with improved CSS scores.

**Discussion:**

These findings support the role of PUM stimulation in mitigating pathological neural complexity alterations, with potential implications for preserving both consciousness and cognitive function in epilepsy.

**Conclusion:**

Further studies are needed to confirm these findings in larger cohorts and to explore the long‐term effects of thalamic deep brain stimualtion in drug‐resistant epilepsy as well as the interaction between neural complexity, awareness, and cognition in this context.

**Key points**

- Stimulation of the medial pulvinar nucleus reduces the loss of brain signal complexity during temporal lobe seizures.
- Preserved brain signal complexity is associated with better awareness during seizures.
- Measuring signal complexity can help track changes in consciousness linked to thalamocortical brain network activity.
- Pulvinar deep brain stimulation may help preserve cognitive function by preventing excessive disruption of brain network organisation during seizures.

## 1 INTRODUCTION

Loss of consciousness/awareness (LOC) is a frequent characteristic of temporal lobe seizures (TLS), significantly affecting quality of life.^1^ When curative‐aim epilepsy surgery is not possible, thalamic neuromodulation (deep brain stimulation—DBS) offers a promising alternative. The medial pulvinar nucleus (PUM) is a potential target.^2^ PUM plays a crucial role in ictal LOC via thalamocortical synchrony.^3^ Acute high‐frequency PUM stimulation has been shown to reduce seizure and LOC severity^4^ and to decrease synchrony, particularly in patients with improved awareness.^5^ Moreover, El Youssef et al.^1^ showed that decreased SEEG signal complexity correlated with LOC in TLS. In this study, we used permutation entropy (PE) to investigate how high‐frequency PUM stimulation alters SEEG signal complexity, in relation to TLS dynamics and ictal LOC.

## 2 METHODS

PE was quantified on the SEEG recordings of eight consecutive patients with temporal focal drug‐resistant epilepsy (fDRE), each having PUM electrode sampling and SEEG‐recorded stimulation‐induced seizures (data set of Filipescu et al).^4^ During a first stimulation session, seizures were induced by hippocampal low (1 Hz) or high‐frequency (50 Hz) stimulation, without additional ipsilateral PUM stimulation (PUM seizures). A second session, performed at least 4 h later, involved inducing seizures by re‐stimulating the same hippocampal‐sampling contacts, this time with additional ipsilateral PUM stimulation delivered as soon as possible after seizure onset (PUM+ seizures). All patients provided written consent; the study was approved by the local ethics committee (PADS25‐142).

Pre‐SEEG cerebral T1‐weighted MRI scans were employed for anatomical segmentation and Virtual Epileptic Patient atlas‐based parcellation (https://ins‐amu.fr/vep‐atlas).^6^ For thalamic segmentation, an in‐house pipeline was used.^7^ All parcellations were then projected into the patient‐specific MRI space. The preimplantation cerebral MRI scan was co‐registered with the postimplantation CT to localize SEEG electrode contacts, which were subsequently mapped onto the VEP atlas—including the thalamic segmentation—using GARDEL software^8^ (https://meg.univ‐amu.fr/doku.php?id=epitools:gardel).

Signal analyses were performed on all nonartifacted SEEG channels, in bipolar montage, using AnyWave software^9^ (https://meg.univ‐amu.fr/wiki/AnyWave). Seizures from the same patient were analyzed using the same montage. PE measures signal complexity by analyzing the distribution of ordinal patterns in a time series. Vectors are created based on an embedding dimension D (number of samples per vector) and a fixed time delay τ (for sample selection). Each vector is converted into a rank‐based ordinal pattern, and the probability distribution of these patterns is used to compute PE, with values ranging from 0 (regular time series) to 1 (random signal).^1^ PE quantification was performed using an in‐house AnyWave Matlab plug‐in and the following parameters: D = 3 samples and τ = 1 sample, using 5‐s sliding windows with 2.5‐s overlap.^10^, ^11^ We further computed Delta Entropy (ΔE), defined as the difference between the minimum ictal and the mean baseline entropy.^1^ Due to SEEG trace curation constraints, the baseline was defined empirically as the 7 s before the stimulated contacts were disconnected from recording. We analyzed matched seizures per patient (PUM− and PUM+ seizures induced using the same hippocampal‐sampling contacts). Furthermore, ictal LOC was assessed using the Consciousness Seizure Scale (CSS).^3^

Statistical analyses were performed using GraphPad Prism. Wilcoxon‐signed rank test was used to assess differences between ΔE values between groups (PUM− and PUM+), with Bonferroni correction applied for multiple comparisons. Spearman's rank correlation was used to examine the covariance between CSS scores and ΔE values.

## 3 RESULTS

We analyzed 19 seizures (11 PUM+ and PUM− pairs): 13 were induced by low‐frequency bipolar stimulation (1 Hz; pulse width: 2000 μs; mean amplitude: 1.41 ± 0.53 mA, range: 0.8–2.5 mA; mean duration: 21.4 ± 16.81 s, range: 6.1–60 s), whereas six seizures were induced by high‐frequency bipolar stimulation (50 Hz; pulse width: 1000 μs; mean amplitude: 1.43 ± 0.35 mA, range: 0.8–1.8 mA; mean duration: 4.67 ± 0.47 s, range: 4–5 s). Pulvinar bipolar stimulation was applied using the following parameters: 130 Hz, pulse width: 450 μs, mean amplitude: 1.44 ± 0.5 mA, range: 1–2 mA, and a mean duration of 5.8 ± 1.23 s, range: 3.16–6.94 s. Individual stimulation parameters and CSS scores have been summarized in Table 1.

**TABLE 1:** Clinical and stimulation parameters of the stimulation‐induced seizures.

Patient ID	Seizure type (with or without additional PUM stimulation)	Hippocampal stimulation parameters				PUM stimulation parameters				Electrographic seizure duration (s)	CSS score
		Frequency (Hz)	Pulse width (μs)	Amplitude (mA)	Duration (s)	Frequency (Hz)	Pulse width (μs)	Amplitude (mA)	Duration (s)		
P1	PUM+	50	1000	1.8	5	130	450	1	5.55	100	5
	PUM–	50	1000	1.8	5	NA				65	4
P2	PUM+	1	2000	2	15.6	130	450	1	5.41	36	0
	PUM–	1	2000	2	14.1	NA				50	0
P3	PUM+	50	1000	1.5	4	130	450	1	3.16	65	1
	PUM–	50	1000	1.5	4	NA				67	4
P4	PUM+	1	2000	1	19	130	450	2	7.7	32	0
	PUM–	1	2000	1	19	NA				25	0
P4bis	PUM+	1	2000	1	19	130	450	2	7.7	32	0
	PUM–	1	2000	2	21.2	NA				113	9
P5	PUM+	1	2000	1	60	130	450	2	6.94	52	0
	PUM–	1	2000	1	11.4	NA				33	3
P5bis	PUM+	1	2000	1	60	130	450	2	6.94	54	0
	PUM–	1	2000	1	11.4	NA				33	3
P6	PUM+	1	2000	1.5	15.1	130	450	2	5.54	74	8
	PUM–	1	2000	1.5	13.3	NA				92	9
P7	PUM+	50	1000	1.2	5	130	450	1	5.56	35	0
	PUM–	50	1000	0.8	5	NA				32	0
P7bis	PUM+	50	1000	1.2	5	130	450	1	5.56	35	0
	PUM–	1	2000	1	13.1	NA				27	0
P8	PUM+	1	2000	1	14.2	130	450	1	5.41	151	4
	PUM–	1	2000	2.5	6.1	NA				138	4

Abbreviations: CSS, Consciousness Seizure Scale; PUM, pulvinar medialis thalamic nucleus.

**FIGURE 1:**
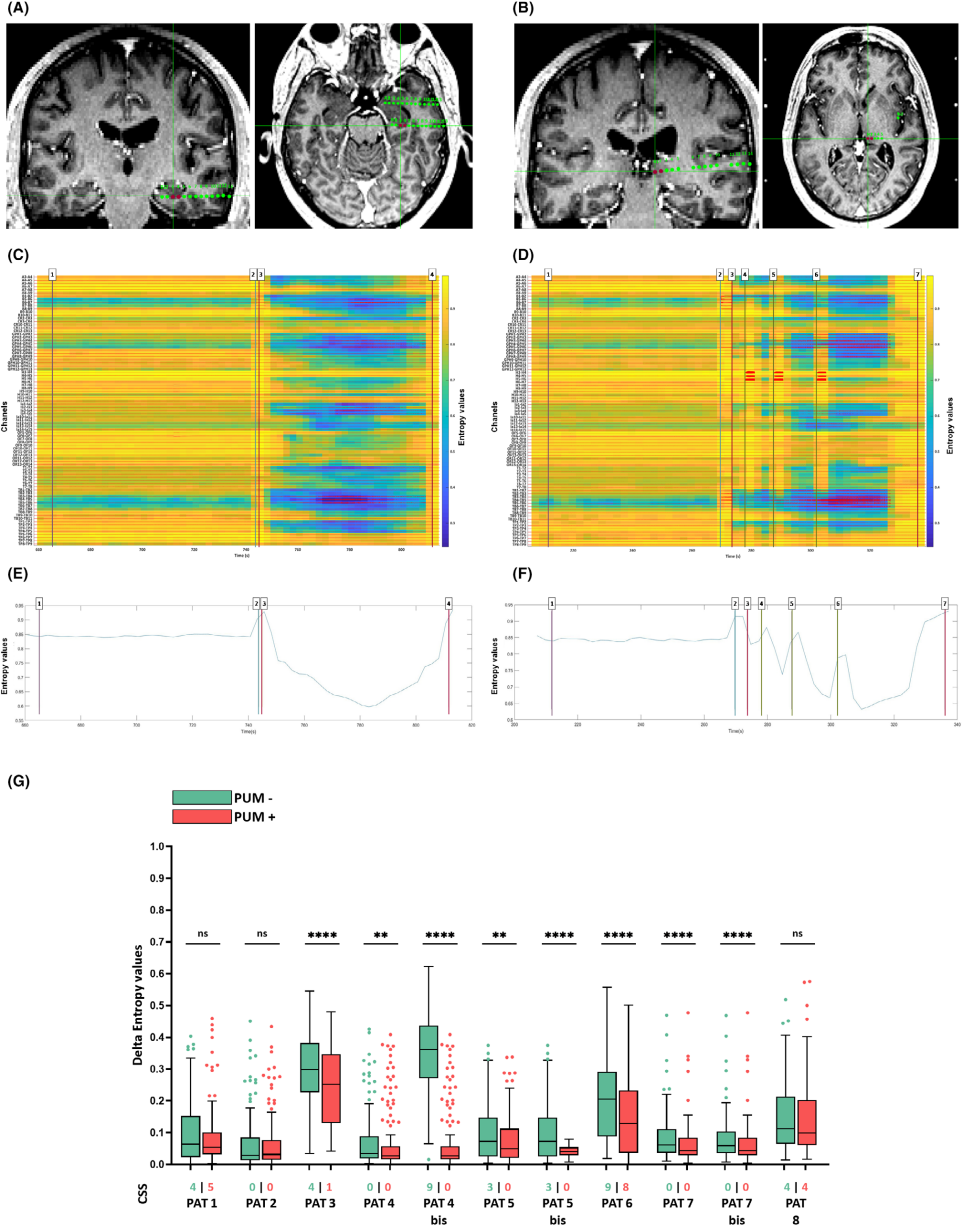
Complexity changes in hippocampal electrical stimulation‐induced seizures without and with ipsilateral pulvinar nucleus ictal stimulation. (A–E) Case example (A, B) Cerebral MRI T1‐weighted MPRAGE sequence—coronal and cross sections with overimposed reconstruction of the electrodes exploring the hippocampus (A) and the ipsilateral pulvinar nucleus (B). The contacts stimulated in each case are marked with red dots: B3‐4 for the hippocampus and H1‐2 for the pulvinar. (C, D) Heatmaps showing the trends of complexity changes during the ictal and peri‐ictal periods in PUM– (without pulvinar stimulation) (C), and PUM+ (with pulvinar stimulation) seizures (D). Dark blue stands for highly organized signal, corresponding to low entropy values, whereas bright yellow stands for chaotic signal, corresponding to high entropy values. (E, F) Global complexity trend as derived from all the analyzed channels. The vertical bars indicate in (C and E): (1) the ending of the baseline period, (2) start of the stimulation that induced a seizure, (3) seizure onset, and (4) seizure termination. The vertical bars indicate in (D, F): (1) the ending of the baseline period, (2) start of the stimulation that induced a seizure, (3) seizure onset, (4–6, and) start of the pulvinar stimulation pulses and (7) seizure termination. (G) Boxplot graph revealing the complexity changes of the SEEG signal as exhibited by the alterations in Delta Entropy values in the two conditions for the pairs of corresponding seizures: Hippocampus electrical‐stimulation induced seizures without (PUM–) and with (PUM+) ipsilateral pulvinar nucleus ictal stimulation. Individual dots stand for Delta Entropy values of each pair of bipolar contacts. Each box has been associated with the Consciousness Seizure Scale score of that seizure. PUM– seizures are figured in green, whereas PUL+ seizures are figured in red. The labelling of the patients includes “bis” if the comparison between corresponding PUM– and PUM+ seizures includes either a PUM– or a PUM+ seizure used in another comparison for that patient. ^ns^ *p* > 0.05; **p < 0.01; ****p < 0.0001 after Wilcoxon comparison with subsequent Bonferroni correction.

Five PUM+ seizures showed an improvement in CSS scores compared to their PUM– counterparts, whereas five PUM+ exhibited no difference with PUM–, and one pair showed a one‐point aggravation in the PUM+ seizure relative to the PUM– seizure (Figure 1G). Visual analysis of the heatmaps and of the global entropy curve, representing the mean global complexity across all exploring contacts in the analyzed montage—revealed the effects of pulvinar stimulation on ictal complexity dynamics. Pulvinar stimulation was associated with one or more increases in signal entropy, coinciding with the timing of pulvinar stimulation (Figure 1A–F). Δ*E* values were significantly lower in PUM+ seizures compared to PUM− seizures (*p* < 0.05 in 9 out of 11 seizure pairs or five out of eight patients). In the remaining cases, although statistical significance was not reached, a similar trend was observed (Figure 1G). A positive Spearman correlation was found between the change in CSS scores (CSS PUM+ − CSS PUM−) and the change in ΔE values (ΔE PUM+ −ΔE PUM−) (ρ = 0.77, *p* = 0.0055).

## 4 DISCUSSION

To summarize, our study demonstrated that a greater reduction in complexity alterations in PUM+ seizures compared to PUM− seizures was associated with reduced LOC. However, this relationship was not consistent across all patients, likely due to interindividual variability and the small sample size. As such, our findings support a possible link between thalamic modulation of SEEG complexity and consciousness preservation, but further studies in larger cohorts are required to confirm this association and to assess the temporal evolution of LOC during seizures. The association between reduced entropy (ΔE) and improved LOC likely reflects a decrease in thalamocortical synchronization, a mechanism previously linked to ictal LOC; however, further studies directly comparing entropy‐based and connectivity‐based measures are needed to substantiate this hypothesis.^3^ By modulating thalamocortical interactions, PUM stimulation may help preserve neural complexity and reduce the severity of LOC during seizures. Similar restauration of EEG cortical complexity and consciousness has been observed in a monkey LOC model.^12^ Our findings align with the growing evidence supporting the role of PUM in seizure modulation and its potential as a DBS target in fDRE.^4^ The PULSE trial revealed decreased seizure severity and improved quality of life after 1 year of PUM DBS, with stable or increased cognitive performance in most patients.^2^ Ictal LOC disrupts information processing and memory formation, contributing to cognitive deficits.^13^ The transient breakdown of network complexity observed in impaired awareness states parallels findings in disorders of consciousness, where decreased neural complexity correlates with reduced cognitive capacity.^14^ By modulating thalamocortical interactions and preserving SEEG complexity, PUM stimulation may not only improve LOC but also protect cognitive function by preventing excessive network desynchronization.

## AUTHOR CONTRIBUTIONS

Ionuț‐Flavius Bratu: Conceptualization, Data curation, Formal analysis, Investigation, Methodology, Project administration, Software, Validation, Visualization, Writing—original draft, Writing—review and editing; Nada El Youssefa: Data curation, Formal analysis, Investigation, Methodology; Stanislas Lagarde: Data curation, Investigation, Methodology; Fabrice Bartolomei: Conceptualization, Data curation, Formal analysis, Funding acquisition, Investigation, Methodology, Project administration, Resources, Supervision, Validation, Writing—original draft, Writing—review and editing.

## CONFLICT OF INTEREST STATEMENT

The authors declare no conflict of interest to disclose.

**Test yourself**

1. What neurophysiological mechanism is most strongly associated with impaired consciousness during temporal lobe seizures?
  - Increased hippocampal entropy
  - Decreased seizure duration
  - Increased long‐distance cortical–subcortical synchronization
  - Loss of thalamic firing
2. What was the main finding regarding ΔE (Delta Entropy) in PUM+ seizures compared to matched PUM− seizures?
  - ΔE values were consistently higher in PUM+ seizures
  - ΔE values were significantly lower in most PUM+ seizure pairs
  - ΔE values showed no systematic differences
  - ΔE was only altered in high‐frequency (50 Hz) stimulation seizures
3. What method was used in the study to quantify signal complexity in SEEG recordings?
  - Fourier spectral analysis
  - Phase–amplitude coupling
  - Permutation entropy
  - Wavelet coherence

## Data Availability

The data that support the findings of this study are available from the corresponding author upon reasonable request.
